# Assessing the distribution of cancer stem cells in tumorspheres

**DOI:** 10.1038/s41598-024-61558-6

**Published:** 2024-05-14

**Authors:** Jerónimo Fotinós, María Paula Marks, Lucas Barberis, Luciano Vellón

**Affiliations:** 1 IFEG-CONICET and FAMAF, Universidad Nacional de Córdoba, Córdoba, Argentina; 2 Stem Cells Lab, IBYME-CONICET, Buenos Aires, Argentina

**Keywords:** Imaging processing, Tumorsphere assay, Cancer stem cells, Biophysics, Computational biology and bioinformatics, Mathematics and computing, Scientific data, Cancer, Cancer models, Cancer stem cells

## Abstract

Cancer Stem Cells presumably drive tumor growth and resistance to conventional cancer treatments. From a previous computational model, we inferred that these cells are not uniformly distributed in the bulk of a tumorsphere. To confirm this result, we cultivated tumorspheres enriched in stem cells, and performed immunofluorescent detection of the stemness marker SOX2 using confocal microscopy. In this article, we present an image processing method that reconstructs the amount and location of the Cancer Stem Cells in the spheroids. Its advantage is the use of a statistical criterion to classify the cells in Stem and Differentiated, instead of setting an arbitrary threshold. Moreover, the analysis of the experimental images presented in this work agrees with the results from our computational models, thus enforcing the notion that the distribution of Cancer Stem Cells in a tumorsphere is non-homogeneous. Additionally, the method presented here provides a useful tool for analyzing any image in which different kinds of cells are stained with different markers.

## Introduction

Cancer Stem Cells (CSCs) are defined by their capability to replicate making exact copies of themselves, or to differentiate giving rise to phenotypically diverse cells. They are resistant to conventional anti-cancer treatments, being thus implicated in disease recurrence and metastasis^1,2^. One of the main barriers to the development of CSCs-targeted therapies is their scarcity in vivo, limiting their availability as experimental systems for pharmaceutical development, and raising the need for production at a scale large enough to fulfill academic and industry requirements. A common solution is the anchorage-independent growth of cancer cells to generate a 3D culture of epithelial cells^3^ enriched in cells with CSCs-like properties such as resistance to anoikis^4^. Thus, and taking into account the limitations of this assay^5^, the resulting tumorspheres are formed by the clonal expansion of a single cell, instead of the self-aggregation of existing cells.

Still, routinely used cell culture techniques are material- and labor-consuming tasks that generate a great amount of inter-culture variability and contamination risks. Moreover, traditional cell culture at a large scale is also cost-ineffective in terms of the high investment in cell culture media and growth factors. In this context, developing forefront, high-throughput screening platforms to identify cytotoxic inhibitors and/or differentiation-promoting agents targeting CSCs becomes of paramount importance. This would require the optimization of a series of bioprocesses that enable the massive culture of undifferentiated cancer cells and the analysis of high volumes of data.

One critical downstream part of such bioprocesses is to assess the cellular response in terms of viability and/or stemness markers, which requires external software for image analysis and segmentation to quantify the relative differences among treatment groups. Even though high-throughput cytometric methods have been developed^6^, there is still the need to know the exact identification and location of putative CSCs. This would help the modeling of CSC dynamics, allowing the development of cost-effective and predictive tools for the examination of tumor evolution and response to therapy. In this regard, mathematical modeling has contributed successfully to the study of tumor growth and treatment response. Indeed, mathematical methods previously developed by us allowed to estimate the expected fraction of CSCs for tumorspheres in different culture conditions^7–9^ and the effect of specific therapies on their development^10^ among other theoretical results^11–14^. In particular, we computationally simulated the growth of a colony of cells in two dimensions using an Agent-Based Model (ABM) that mimics basic features of CSCs proliferation to form a spheroid^15^. The simulated spheroid grows from a single CSC and the cells can undergo mitosis at a fixed rate (the Population Doubling Time or PDT). Depending on the intrinsic and extrinsic (micro-environment) signals, the CSC will replicate generating another CSC with a certain probability $p_s$, yielding a *differentiated cancer cell* (DCC) otherwise. This simplified model allowed us to estimate the total number of CSCs, the fraction of CSCs situated on the periphery of the colony, and the size of the whole spheroid, showing that these traits are dependent on the replication probability ($p_s$) of the CSCs. Indeed, simulating with intermediate replication probabilities, we observed active CSCs at the border of the colony and detected that they form a path that links the center of the colony with its border. Furthermore, an increase in the replication probability led, as expected, to a large CSCs population that overtook the system. This last situation may describe most experimental conditions used for culturing tumorspheres^3,16–19^ and agrees with our previous mathematical models^7–9^. Inspired by these simulations, in the present work we generate tumorspheres from MCF-7 cells and analyze them by confocal microscopy to obtain a method of image processing that fits biological data with already developed mathematical models for the distribution of CSCs in tumorspheres^15^. We chose tumorspheres as a simplified 3D tumor model since they are cellular structures generated from a variety of tumors from epithelial tissues, such as breast, lung, prostate, or colorectal cancer^20^. Even though the optimal conditions for culturing tumorspheres may differ among tumor types, this experimental system can recapitulate the physicochemical gradients from the spheroid periphery to its core and mimic, to some extent, mechanical properties and cell-cell interactions of avascular tumor mass microregions^20,21^. Here, following a Data Science approach, we have developed an advanced computational method to detect the expression and distribution of the stem-like cells, SOX2-positive cells, under the assumption^18^ that this stemness factor is expressed in tumorspheres from cell lines and primary cultures. The processed images are used to study the distribution patterns of CSCs, employing statistical tools that validate our main computational finding: that CSCs are heterogeneously distributed in a tumorsphere.

In summary, we report a protocol to analyze confocal images of tumorspheres, stained with a stemness marker, to statistically infer the more suitable threshold to determine which cells in the culture belong to the stem phenotype. We highlight the capability of our method to extract data from the experiments, allowing direct comparison with simulated cultures. As an example, we show the detection of a non-homogeneous distribution of CSCs in tumorspheres as predicted with a previously developed computational model.

## Results

According to simulations^15^, CSCs must be connected in tumorspheres forming “paths” that join the center of the spheroid with its border. To assess this, we performed tumorspheres assays where MCF-7 cells in suspension cultures proliferate in a solution enriched in growth factors to ensure a large fraction of CSCs. After 9 days of growth, we collected the spheroids and attached them to a slide by cytocentrifugation. Due to the centrifugal forces, their spheroidal shape may be lost, becoming discs smashed against the slide, however, the structure of the tumorsphere is maintained. We stained the slides to detect the location of all cellular nuclei (DAPI), and the corresponding primary and secondary antibodies needed to detect the position of the stem cells (SOX2). Finally, we took pictures on several focal planes (*slices*) of the spheroid obtaining a full 3D reconstruction. Because of the smashing of the spheroids, only one or two slices of the image were needed to observe all the cells present in them. These images were processed with computer vision and statistical analysis techniques, among others. The result of the process allows us to mathematically reconstruct the discs, specifying the position of all their cells, marking those candidates to be CSCs and statistically deciding which ones are truly CSCs. The experiment and the image processing procedure are fully detailed in the “Methods” Section.

### The cancer stem cells distribution

**Figure 1:**
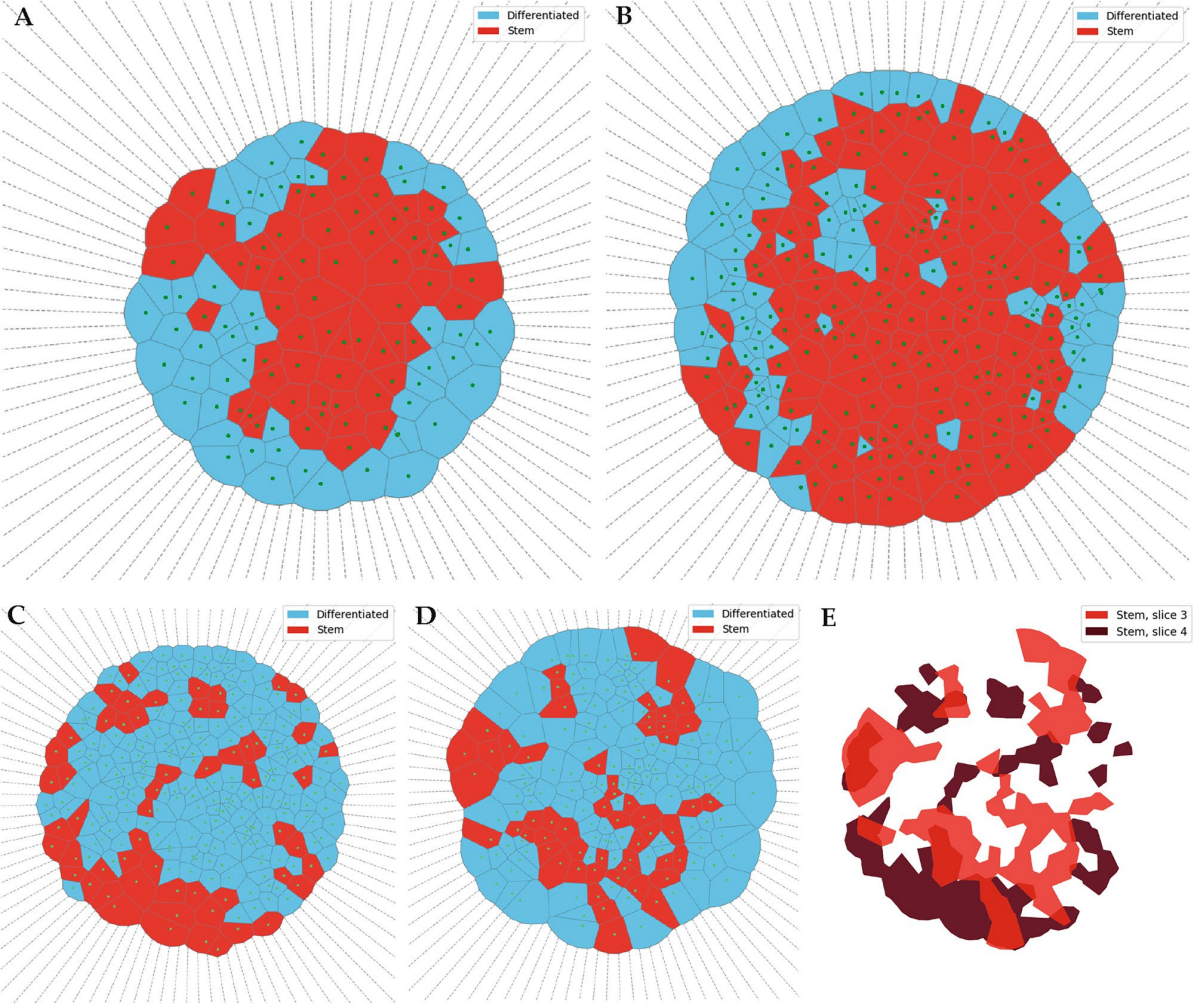
Reconstruction of the spheroids’ slices after image processing. For different slices (i.e., focal planes) of the experimental spheroids, the cell’s area is approximated by polygons. The polygons representing CSCs are colored in red, and the ones representing DCCs, are colored in blue. Note that the CSCs are not uniformly distributed in any image, and form patches at the border of the aggregate. The panels correspond to: (**A**) Sph4, slice 2; (**B**) Sph3, slice 3; (**C**) Sph1, slice 3; (**D**) Sph1, slice 4; (**E**) Overlay of CSCs of slices 3 and 4 of Sph1. The places where the two slices overlap are depicted in a third intermediate color to highlight how stem cell clusters are connected through them.

Our main results are summarized in Fig. 1, in which we can observe the distribution of the two cell phenotypes: CSCs in red and DCCs in blue. We present three representative examples belonging to the reconstruction of three different spheroids. After the filtering and reconstruction process of the confocal images, we obtained pictures of the cell’s distribution as a Voronoi tessellation (see “Methods” Section). That is, each cell is represented, in Fig. 1, as a polygon whose centroid coincides with the centroid of the corresponding cell in the pictures. Note that this representation is just an approximation of the shape of the cell that allowed us to quantify the SOX2 content inside the cell and decide, using statistical tools, if the cell displayed a stem or a differentiated phenotype. After the whole process, the CSCs become represented by red polygons and the DCCs by blue polygons.

#### Patches in the border

Our first example, labeled Sph4, slice 2 as in raw data files, a spheroid that has not grown much is depicted in Fig. 1A. Considering the different proliferative capacities of the cells in these culture conditions and the possibility of differential access to the growth factors, we expect inter-spheroid variability in both the number of cells and the CSC fraction. In this case, the spheroid had less than a hundred cells, with half of them being CSCs, cf. Table 1. There is an evident higher concentration of the CSCs in the core of the spheroids, which could be explained by the model used in our simulations: the first CSC has a given chance of generating another CSC, but when one of these CSCs differentiates the first time, its lineage will only contain DCCs that will surround its stem mother. The only exception is when a DCC cannot undergo mitosis in a nearby place, already occupied by a CSC. Indeed, this CSC will continue generating other CSCs until one of them becomes differentiated, leaving in the process a path of CSCs among DCCs. This is exactly what is shown in panel A of Fig. 1: at an early stage, red CSCs become surrounded by blue DCCs in the lower portion of the spheroid. However, three CSCs were able to initiate paths that extend to the periphery of the spheroid. The result is that CSC will form patches in the border of the spheroid.

The spheroid shown in Fig. 1B, labeled Sph3, slice 3, has more cells in total and more CSCs than the one in panel A, but its fraction, 64%, is larger than the 54% of the previous case. This result agrees with our probabilistic modelization for the outcome of the mitosis of a CSC^9^.

In this example, we can also appreciate the deformation produced by the smashing against the slide. Some DCCs appear in the bulk of the disk surrounded by CSCs. These DCCs could have initially belonged to the border of the spheroid and reached the center after centrifugation. In this manner, it is easy to understand why cells closer to the center of the disk could be a mix of original center cells and some of the periphery. However, it is very unlikely that cells at the border of the spheroid could originally be from near the spheroid center.

#### Two layers sample

As mentioned, our method for placing the sample on the slide alters the shape of the spheroids, projecting the cells onto a disc. Depending on the forces acting on the spheroids, sometimes their cells can not push all their neighbors apart, piling up on top of them. As a consequence, we need to use the images of more than one slice to recover all the cells that constitute the spheroid.

An example of this is given by Sph1 which had two layers of cells superimposed and required two slices of confocal imaging to access all the cells. In panels C and D of Fig. 1 we depict the obtained cell distribution for the upper and bottom layers respectivelly. As observed by direct inspection, and reported in Table 1, this spheroid has a larger amount of cells than those in the previous examples, but a much smaller CSC fraction. Thus, even though CSCs are thought to drive tumor growth, their abundance may not be directly proportional to it. This can be understood through our modeling hypothesis about CSC reproduction. When CSCs have a small chance to replicate becoming differentiated at early times, they are likely to become quickly surrounded by DCCs. This is again consistent with our description of the replication rate as a probability that models the chances of a CSC becoming undifferentiated either because of extrinsic or intrinsic causes.

In the two-layer disc, we have an example of such a situation, where it becomes difficult to follow the CSCs’ path. The CSCs seem to be less connected and not as clustered as in the previous cases, and might even seem more randomly distributed. Nevertheless, this case presents the opportunity for a better reconstruction of the spheroid due to the smaller loss of spatial information. Indeed, if we subtract the DCCs from the snapshots in panels 1C and 1D, and overlay the remaining CSCs, we obtain the distribution shown in panel 1E. Note that we colored the bottom layer cells (against the plate) in dark red, and the upper layer in light red. The result is now evident, most of the CSCs are indeed connected, forming a path along the two layers as we expected from the simulations.

**Table 1:** Experimental size and composition of the spheroids, recovered after processing the images.

	Sph4, slice 2	Sph3, slice 3	Sph1, slice 3	Sph1, slice 4	Sph1, both slices
Radius (µm)	87.58	102.04	113.25	99.07	–
Total cells	112	240	183	252	435
Stem cells	59	155	55	56	111
Differentiated cells	53	85	128	196	324
Stem cell fraction	53%	64%	30%	22%	26%

For each slice, we report the radius of the spheroid, the total number of cells (as well as the number for each population), and the fraction of them that are stem cells. In the case of Sph1, we reported the measured values for each slice and the total values, summing up the data of the two slices in the last column. Note that this spheroid, being the larger one, has the smallest CSC fraction, in agreement with the simulations.

### Non-randomness of the CSC’s distribution

**Figure 2:**
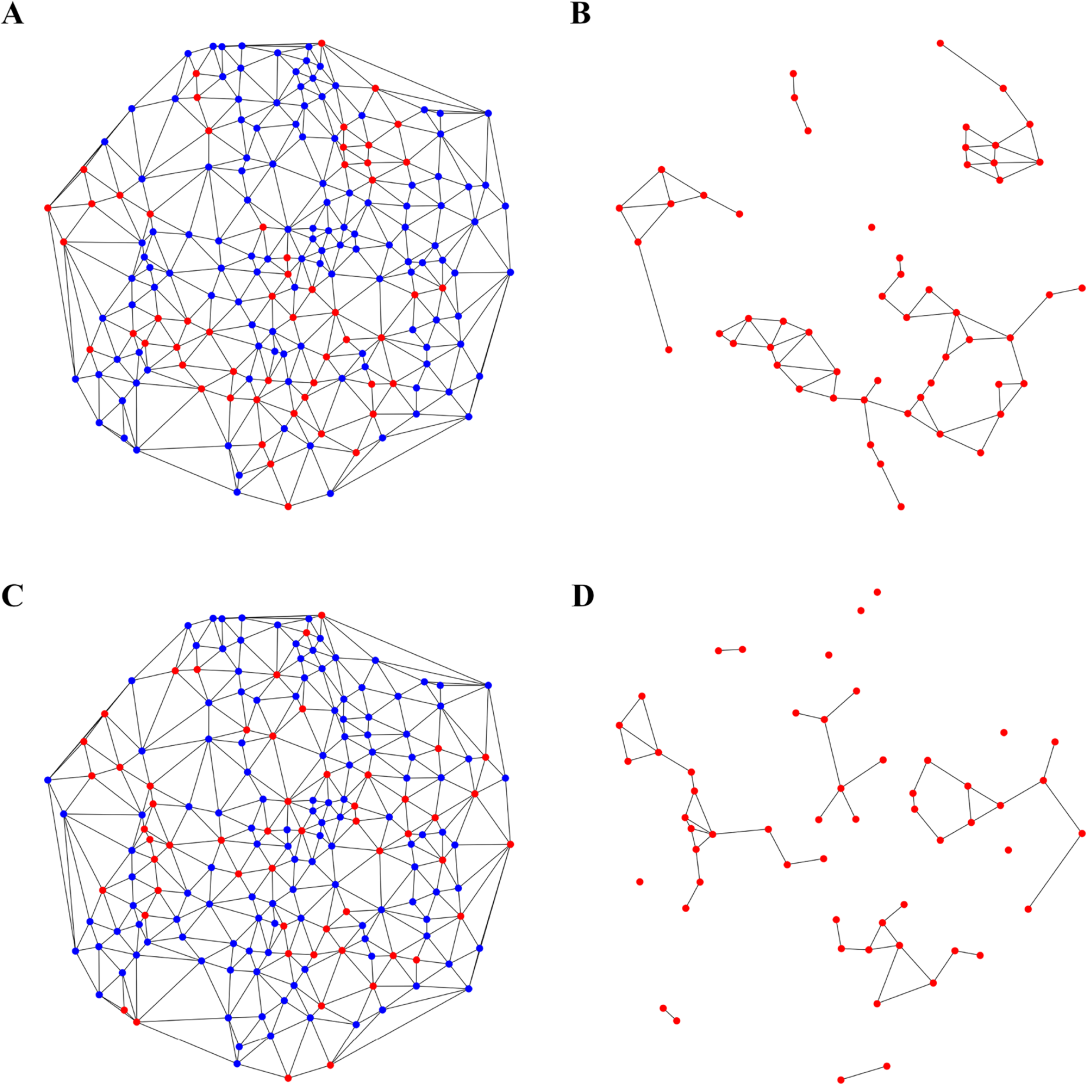
Comparison of an experimental network and its stem subgraph, with a copy of it with randomly located CSCs. (**A**) The reconstructed network for Sph1, slice 3 with its CSCs plotted as red dots, and its DCCs plotted as blue dots. Neighbor cells are connected with gray links. (**B**) The CSCs subgraph corresponding to the network in A is obtained by deletion of the DCC (blue) nodes. The number of stem-connected components is 5. (**C**) The random graphs are constructed by randomly redistributing the red and blue dots on the same network. (**D**) The CSCs subgraph corresponding to the randomized network in C has a different number of connected components, 13 in this case. In most random cases, this number is larger than in the experimental network.

To quantify our qualitative observation that CSCs are not uniformly located in the spheroid, we measure extra features of each spheroid. The tessellation generated by our method defines the location of a cell and its neighbors, allowing to make a graph of the connections between cells. An example of this is depicted in Fig. 2A for Sph1, slice 3, where the CSCs are represented by red dots, and DCCs by blue dots. If two cells are neighbors, they are linked with a black line. With this graph, we can use network theory to measure how connected are the CSCs inside the spheroid structure.

At first glance, It is easy to see that CSCs form paths as expected. Moreover, we assessed if this is true by measuring how many of these paths are present in the graph and, most importantly, how apart one is from the other. The physics of complex systems has developed tools to analyze graphs and collect nontrivial information. In our case, we determine if the paths formed by CSCs could appear by chance or if there must be a defined mechanism behind them. As explained in the “Methods” Section, we test for homophily in the reconstructed networks for the experimental systems. That is, the tendency of cells to neighbor other cells of the same phenotype. To characterize this, we use the *assortativity coefficient*, which is greater than zero for networks that present homophily, and results positive for our experimental cases. Also, the *homophily ratio*, the fraction of pairs of neighbors of the same kind, gives relatively large values (meaningful comparison to be established later). Both results mean that the nodes prefer to be attached to nodes of the same kind. The measured values of these quantities for the four examples shown here, are reported in Table 2 under the columns labeled ‘experimental’.

For a meaningful comparison and interpretation of these values, we used a classical approach from the physics of complex systems. We maintain the connections between the nodes of the previously obtained networks, but this time we arbitrarily set which ones are the CSCs, keeping their total number equal to the corresponding experimental network, cf. Fig. 2C. These random distribution of the CSCs is one of the most used hypotheses in mathematical modeling. This will also resemble the case of CSCs that can travel inside the spheroid. We obtained 10000 of these networks with randomly distributed CSCs, and we calculated the quantities mentioned before for characterizing their homophily. Then, we performed statistical tests (always using significance $\alpha _t=0.001$) to compare these values with the ones obtained for the experimental cases. These tests tell us whether the quantities measured in the experimental networks significantly differ from the same quantities measured in the random networks. In Table 2 we reported the averaged results over all the realizations including their standard deviation. The assortativity coefficient is now zero meaning that, as expected, there is no preferential attachment of cells of the same kind when we randomly locate the CSCs. The random homophily ratio is a bit larger in the two-slice spheroid but still lower than the experiential case. Thus, experimental values tell us that daughter cells indeed stay close to their parent cell.

A way to double-check this result is by looking at the stem subgraphs. As shown in Fig. 2B, this is the network formed just by the CSCs and the connections between them. We measured the degree distribution of these subgraphs, deriving information such as the average number of connections that the nodes have. We also calculated the number of connected components of this graph, which is the number of separated groups of CSCs. The more clustered the CSCs, the less the connected components. These values are also reported in Table 2 both for the experimental and the average over the random networks.

For Sph3 and Sph4 the large CSC fraction made the experimental and ensemble values statistically indistinguishable from each other. This means that, because we have more than half of CSC in the spheroid, there is no chance to get many isolated CSC, independently of the way they are distributed. On the other hand, for Sph1 the measured number of connected components was significantly smaller than the one measured for the random ensemble, stressing that despite being fewer CSCs than DCCs, they are indeed connected. A close inspection of Figs. 1 and 2 reveal that most of the connected components are separated from each other by just one DCC as predicted by simulations. Thus, the number of *effective* connected paths, in the sense defined in^15^, should be even smaller.

The mean degree of the experimental stem subgraphs was significantly higher than their corresponding random averages. This implies that CSCs are significantly more connected between them than expected if located randomly. This, in turn, reinforces our previous conclusion about the tendency of neighboring cells of the same phenotype.

**Table 2:** Homophily comparison of the experimental and the random ensemble of networks.

	Sph4, slice 2		Sph3, slice 3	
	Experimental	Random	Experimental	Random
Assortativity Coefficient	0.39	− 0.010 ± 0.04	0.47	− 0.01 ± 0.06
Homophily Ratio	0.74	0.50 ± 0.03	0.73	0.54 ± 0.02
Stem Connected Components	2	3.7 ± 1.5	2	1.1 ± 1
Degree of Stem Subgraph	4.5	3.0 ± 0.2	4.8	3.7 ± 0.1

	Sph1, slice 3		Sph1, slice 4	
	Experimental	Random	Experimental	Random
Assortativity coefficient	0.22	− 0.01 ± 0.04	0.26	− 0.01 ± 0.01
Homophily ratio	0.67	0.58 ± 0.02	0.74	0.65 ± 0.01
Stem connected components	5	17 ± 3	12	25 ± 3.3
Degree of stem subgraph	2.7	1.7 ± 0.2	2.64	1.3 ± 0.2

Network properties from the experimental networks are shown in columns labeled ‘experimental’, and the ones for networks with randomly distributed CSCs, in columns labeled ‘random’. For these last ones, we report the mean values, over 10,000 runs, and their standard deviation. The assortativity coefficient is zero for the random cases, stressing that there is a no preferential attachment, contrary to what happens in the experimental cases where positive values are always found. For Sph4 and Sph3 the connected components are similar due to the large amount of CSCs.

## Discussion

As suggested by simulations, CSCs are not randomly distributed in the tumorsphere, they rather form paths and tend to be near to cells of the same type. This anticipation stems from the fact that, while we expected the two-dimensional simulations to yield different quantitative results than three-dimensional systems, the qualitative behavior will likely remain the same. The CSCs would be heterogeneously distributed and connected by ‘paths’, creating “patches” at the border (surface) of the spheroids after approximately one week. This connectivity might reflect the stem cell competition. Stem cells reside within specific microenvironments (niches) which provide restricted maintenance signals and limited physical space, and consequently, stem cells are constantly competing with their neighbors for niche occupancy^22^. In the non-neutral stem cell competition, a fraction of stem cells gains a fitness advantage or disadvantage over their neighboring stem cells. Thus, the more competitive fraction overtakes the niche, sometimes disrupting it and leading to diseases such as cancer^23^. Indeed, mutations that affect cell fitness either in development or homeostasis can lead to a competitive growth advantage and potentially clonal expansion. Alternatively, non-neutral stem cell competition eliminates “unfit” clones, for instance when aneuploid cells are depleted during development^24^. However, in the case of tumorspheres, it is difficult to assume the type of stem cell competition due to the selection already imposed by the culture conditions. It is possible, however, that the hypoxic cores in the tumorsphere can trigger differential cell responses leading to drug resistance^25^ and these CSCs, more competitive, deplete the drug-sensitive cell populations and eventually reach the surface of the spheroid through replication. One example of this has been reported in tumorspheres from the breast cancer cell line BT474, in which upregulation of HER2 expression led to a hypoxia-conditioned breast CSCs population with increased resistance to trastuzumab^26^. Even though these effects have been observed in large-sized, multicellular spheroids with significant oxygen, nutrients, and metabolite gradients, the fact that small spheroids (25–50 cells) also contain cells more resistant than monolayers to chemotherapeutic agents has been repeatedly observed long ago^27^. This suggests that other factors, possibly mechanical or geometrical restraints imposed by the shape of the tumorsphere, affect CSCs location and cell-cell interactions. Therefore, mathematical models of how different subpopulations of cells interact in the context of a multicellular aggregate of spheroidal shape and validation of these models with biological data may contribute to developing cost-efficient and predictive tools. In this regard, the information that imaging can provide is highly relevant for setting up simulations, formulating mathematical models, and fitting their results.

To assess the frequency and location of CSCs, it is necessary to maintain the structure of the spheroid as a whole, as well as its undifferentiated state. Multicellular aggregates growing in suspension are difficult to attach onto slides. We overcame this issue by cytocentrifugation, although this procedure may induce some deformation on the spheroids, as mentioned in the “Results” Section. Indeed, Leis and coworkers have previously used it to assess SOX2 expression in the whole spheroid^18^. Moreover, in the same work, the authors use e-cadherin staining as a control for dye penetration, also indicating that the integrity of cell-cell adhesions is unaltered. In other work, the same group demonstrates the integrity of cell-cell adhesions following cytocentrifugation^28^. Thus, the deformation of the spheroids does not alter the relative location of the different cell types.

Most of the cytometric methods developed recently quantify spheroids viability and morphology in response to different conditions/treatments^6,29^. In addition, they usually depend on expensive live cell imaging equipment and/or proprietary software packages and do not assess the distribution of CSCs within a spheroid. Some image segmentation methods for automated spheroid quantification^30^ can be performed on images obtained with an inverted light microscope, but still perform multispheroid analyses. We provide open software for the automated and massive analysis of CSCs distribution at a single-spheroid level, thus contributing to fill this need.

It is worth mentioning that all the code that implements the procedure applied to the images presented here is completely open-source, fully automatized and versioned. This intends to not only foster reusability and collaboration, but also to ensure reproducibility. Moreover, not needing to manually carry out the analysis dramatically reduces the amount of time needed to obtain results and provides the scalability required for analyzing whole samples of data. This is essential for carrying out statistics over the sample of spheroids, which will be the subject of future work.

The method for analyzing the images presented here could be improved or modified in several ways to increase its accuracy or be made suitable for other applications. For instance, the denoising process used here was chosen to increase the performance of the neural network on our particular set of images. Processing a different set of images may require adjusting the denoising steps. Another aspect that could be modified is how many groups are found on the images. Changing the algorithm to find a prescribed, fixed number of groups is straightforward. However, in case the number of groups is unknown, or the presence of subgroups is suspected, it is easy to implement more robust ways of finding the appropriate number of clusters. One way to achieve this would be to first select a criterion, for instance, the one given by fitting a GMM for a fixed number of clusters. Then, we would rank the criterion using the Bayesian Information Criterion (BIC) or the Akaike Information Criterion (AIC), for different numbers of clusters. Both these criteria take into account the likelihood of the model, penalizing in different ways its complexity. A simple plot of the BIC and AIC scores as a function of the number of clusters would be enough to spot its minima, which will indicate the appropriate number of groups.

As a final remark, we could also measure both, the total amount of cells in each spheroid, and the respective CSC fraction. Furthermore, we could estimate that the growth rate for these spheroids is around $r=$ 1.1cell/day, which allows us to establish and set the temporal scale of simulations as those in^15^. This value agrees with the one obtained by fitting experimental data with mathematical modeling^9^, and encourages us to increase the complexity of our mathematical and computational models, for example, extending them to three dimensions. Information like this will aid in improving the accuracy of mathematical models as the ones mentioned in the introduction, among others.

## Conclusion

The method of image processing presented here is devoted to recognizing the location of the CSCs in microscopy images. Its originality lies in the fact that we are not just looking at where the SOX2 fluorescence is high enough under a subjective criterion. The key is the use of the Gaussian Mixture Model to fit the data and separate, with a statistical criterion, both cell populations. Having done this, the way of depicting the spheroid or what is done with the resulting data will depend on the questions that researchers have in mind. In our case, we presented an example that compares the experimental CSC distribution in tumorspheres with a previous computational model. Beyond the fact that we are pleased to find that *in silico* and *in vitro* experiments give similar outcomes, the method proposed here is straightforwardly applicable to other similar experiments. Indeed, to definitively assess the CSCs distribution in a spheroid, we must be able to put into the microscope non-deformed spheroids and take pictures of several slices of them. Furthermore, according to our computational simulations, the confidence in our result will exponentially increase with the cultured time. Indeed, we are carrying out full 3D simulations of tumorsphere assays. The preliminary results still support the finding that CSCs are heterogeneously distributed inside the spheroid. However, the probability of finding CSCs on the border of the spheroid seems to be significantly enlarged. These simulations plus the help of confocal images of several slices of a non-deformed spheroid, will be the basis for studying more specific anti-CSCs therapies.

## Methods

### Cell culture and tumorsphere assay

Human breast cancer-derived cell line MCF-7 was maintained in DMEM/F12 complete medium (DMEM F12 + FBS 10 + 1 Glutamine) at 37 °C in a humified 5 CO_2_ atmosphere. The tumorsphere assay was performed according to modifications^18^ from the original protocol by Dontu et al.^3^. Briefly, 6-well plates were treated with poly(2-hydroxyethyl methacrylate) to prevent cell adhesion. MCF-7 cells were seeded at 3000 cells/ml in 3 ml of complete mammospheres medium (DMEM/F12 + B27 2 + glutamine 1 + 20 ng/ml EGF + 20 ng/ml bFGF) per well. Cells were incubated at 37 °C in a humified 5 CO_2_ atmosphere for 7–9 days, adding 0.5 ml of medium with growth factors every 48 h. The resulting mammospheres were collected and attached by cytocentrifugation onto slides for immunofluorescent detection of SOX2.

### Immunofluorescence

Immunofluorescent detection of SOX2 in mammospheres was performed as described in Leis et al.^18^: slides were fixed in methanol and then washed 3 times in washed solution (PBS-BSA 0.1) for 5 min and permeabilized with PBS-BSA 0.1 + 0.3 triton X100 for 30 min at RT. Following permeabilization, the slides were incubated in blocking solution (PBS-BSA 0.1 + Normal Goat Serum) and the corresponding primary antibody (anti-SOX2, cat # PA1-16968, Thermo) ON at 4C. Next, the slides were washed 3 times in washing solution for 5 minutes and incubated with the corresponding secondary antibody (anti-rabbit Alexa Fluor (TM) 555 cat # A31572, Life Technologies).

### Confocal imaging

Images from 3 representative spheroids after carefully scanning the slide were acquired with a Zeiss LSM 880 Laser Scanning Confocal microscope with a 20X objective. The corresponding lasers and photomultipliers for excitation and detection of Alexa Fluor 555 and DAPI were used. Further specifications regarding the obtained images are available at the public repository specified in the Additional information section.

### Image analysis

In the files obtained by digital imaging through confocal microscopy, we recognize the number, position, and extent of cells in the spheroids, and tag CSCs and DCCs according to the amount of SOX2 fluorescence using a statistical criterion. In the following, we describe the whole process and show the partial results on spheroid number 1, Sph1 hereafter, as an example. We began with the corresponding .czi file that contains data on three channels labeled *optic*, *DAPI* with the fluorescence of the nuclei, and *SOX2* with the SOX2 fluorescence. The data are files containing the position of the pixels in three spatial dimensions (*x*, *y*, *z*) with 2292 pixels representing $0.12 \mu \text {m}$ for each side in the horizontal $x-y$ plane, and a thickness of 9 pixels representing $2 \mu \text {m.}$, in the *z* direction. For each pixel, the corresponding value for each channel is included. We start analyzing each channel separately and then, we merge their information.

#### The SOX2 channel

**Figure 3:**
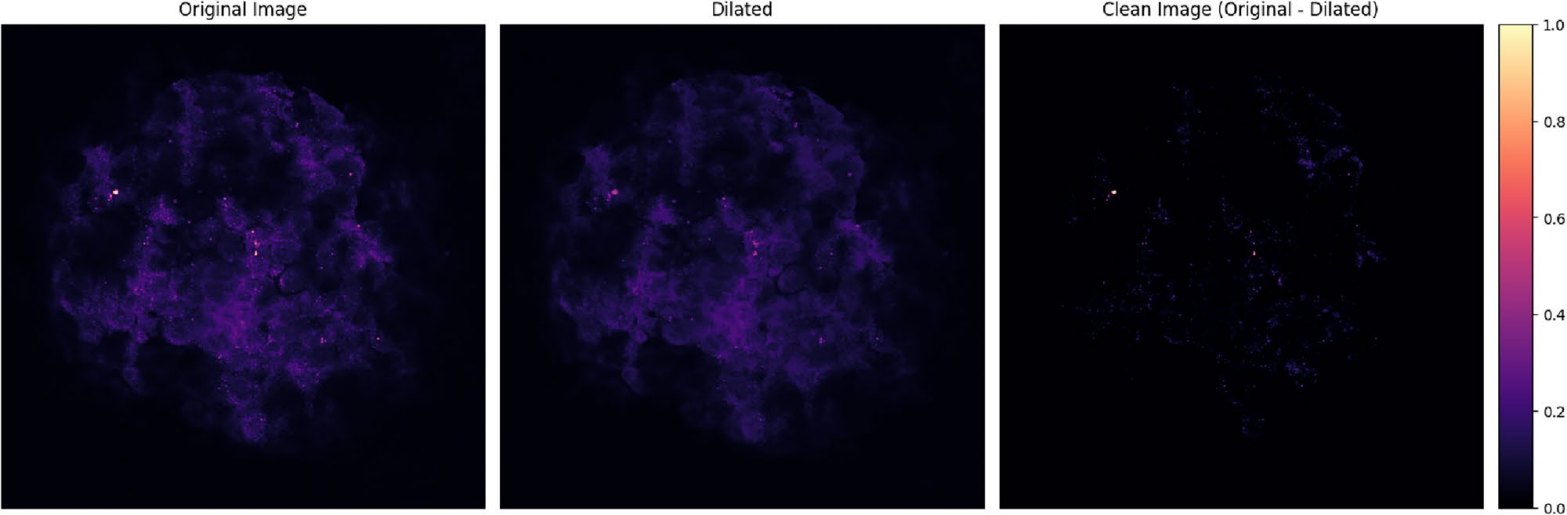
Cleaning of the SOX2 channel. From left to right: the original image, a dilated version of it (a blurred version or background noise), and the cleaned image as the difference between them. The original image corresponds to the SOX2 channel of Sph1, slice3. Note that the dilated image allows us to remove a large amount of non-informative pixels.

The technique reveals the spots where the marker was attached to the cells, getting rid of the diffused SOX2 in the bulk. To achieve this, we perform a reconstruction by dilation, a morphological image processing technique that provides a denoised version of the image by, subtracting from it, a blurred version of the original one. Figure 3 shows the original image in the left panel, the dilated image at the center, and the resulting cleaned image in the right panel. Specifically, we used a Scikit-image’s implementation of the morphology algorithms. For further information, see scikit-image ’s documentation).

#### The nuclei channel

For this channel, we use the nuclei to individualize and identify the cells. Specifically, we performed instance segmentation on the nuclei, extracting geometrical features such as the area and the coordinates of the center of the segmented objects by the following the next steps:

1. Contrast Limited Adaptive Histogram Equalization (CLAHE): Used for local contrast enhancement (see documentation) makes the edge recognition easier for the segmentation algorithm.
2. Morphological processing: To further improve the edge recognition, we performed a morphological opening (erosion followed by dilation), followed by an area closing (similar to a morphological closing, dilation followed by erosion, but using a deformable rather than a fixed footprint). The opening helps separate objects that may be in contact. The area closing removes small dark structures to avoid single nuclei being segmented as many objects due to dark spots within them (see documentation).
3. Bilateral Denoising: An edge-preserving bilateral filter denoises the image, averaging pixels based on their spatial closeness and radiometric similarity. This filter works both to reduce the noise introduced by the morphology operations and by out-of-focus objects (see documentation).
4. Instance Segmentation: For identifying the nuclei, we used the 2D_versatile_fluo model (see documentation). This is a Stardist^31^ convolutional neural network with a U-Net architecture, trained on fluorescence microscopy images similar to the ones of our experiment. We chose this model instead of instance segmentation with bounding boxes because, in this way, we do not need a subsequent shape refinement. Furthermore, semantic (per-pixel) cell segmentation requires a subsequent pixel grouping that can result in segmentation errors such as falsely merging bordering cells. Since the images portray situations of very crowded cells, such errors would be very likely to happen in our case. Star-convex polygons provide a much better shape representation that overcomes these difficulties.

**Figure 4:**
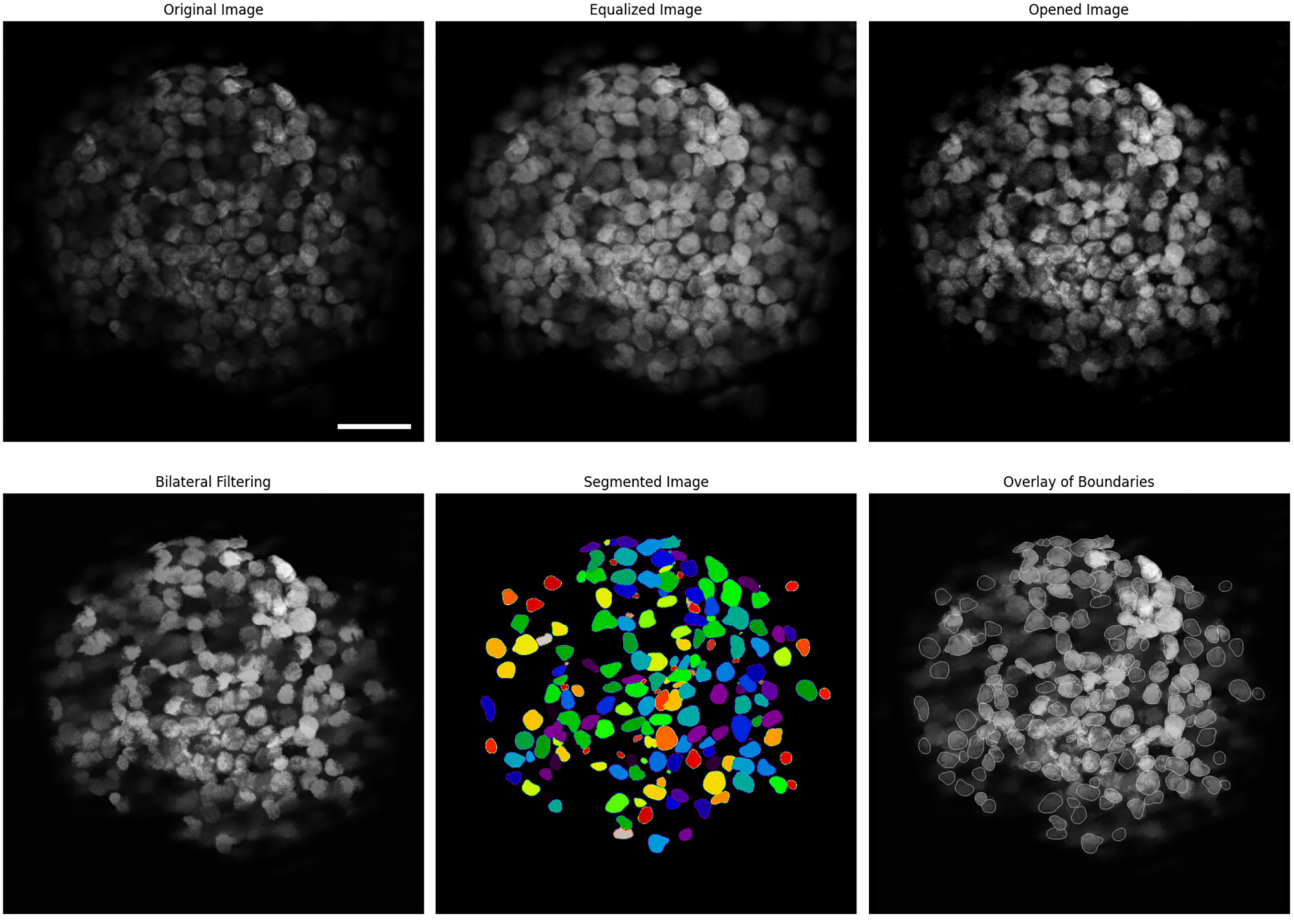
Image processing of the nuclei fluorescence channel (DAPI). From left to right and top to bottom, it is possible to follow the different steps in the processing. In the bottom right corner are shown the borders of the identified structures over the original image. The darker, unrecognized nuclei correspond to cells that are outside, in this case behind, the focal plane. The scalebar in the first picture is $50\mu m$.

The complete process on DAPI channel is depicted in Fig. 4. The original image is in the upper left corner. The lower right image is an enhanced representation highlighting the reconstructed cell boundaries.

#### Tessellation for cell region identification

**Figure 5:**
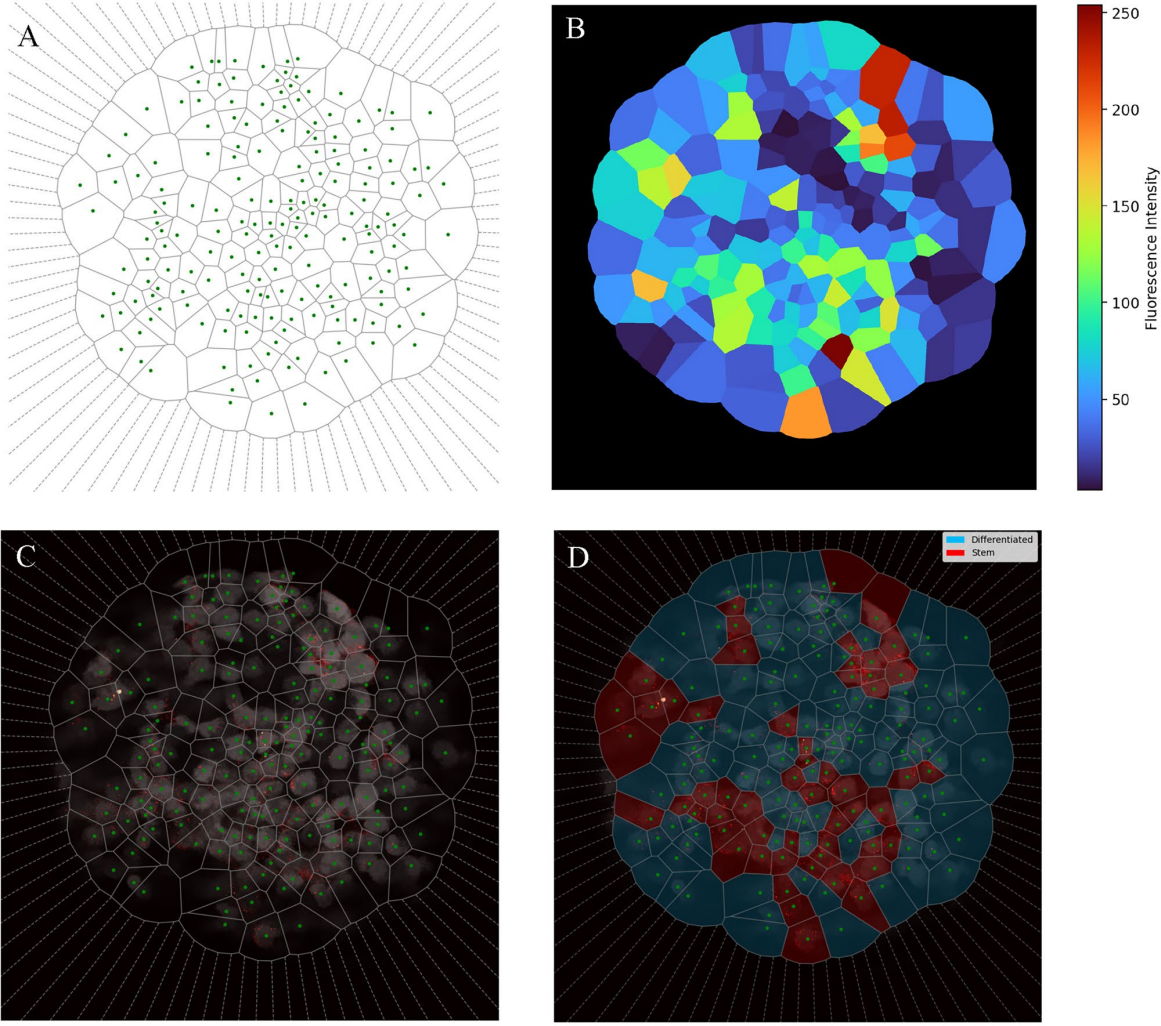
From tessellation to CSC recognition. (**A**) Voronoi tessellation. The centroids of the cells, green dots, were used to estimate the cell area. (**B**) Regions colored according to SOX2 fluorescence intensity. For each region of the tessellation, the sum of its SOX2 fluorescence intensity is computed. (**C**) Tessellation overlaid with nuclei (gray scale) and SOX2 (red palette) channels. The boundaries between Voronoi regions are plotted with gray lines. (**D**) Tessellation regions are colored in red for Cancer Stem Cells and blue for Differentiated Cancer Cells, according to their SOX2 fluorescence content. If the fluorescence surpasses the threshold given by the GMM, the cell is considered a stem one.

Since the SOX2 marker is not necessarily bound to the nucleus of the cell, but rather to its cytoplasm, we need a way of assigning each point in space (i.e., each pixel) to a single cell to further associate the fluorescence of the marker with a given cell. To do this, we approximated the division of the space associated with each cell by a Voronoi tessellation. We used the centers of the segmented objects filtering out the ones corresponding to cells that do not belong to the spheroid when needed. The result is shown in Fig. 5A. It is worth mentioning that we used artificial points, uniformly distributed in a circumference enclosing the spheroid, to limit the area of the cells in its border.

We thus associated to each cell, the sum of the fluorescence intensity of each pixel of the SOX2 channel contained inside its corresponding Voronoi region. The result of this association is shown in Fig. 5 as a color scale depicting the amount of SOX2 in each Voronoi region. To better visualize the tessellation, we overlayed it on the nuclei fluorescence and the cleaned SOX2 channel in Fig. 5C.

#### Stemness threshold in SOX2 fluorescence intensity

To identify the CSCs is needed to define a threshold in the intensity of SOX2 fluorescence that divides non-stem from stem cells. This threshold is usually set by hand but here, we choose a quantitative approach. We implemented a clustering algorithm to group the intensities of the cells and an *elbow plot* using k-means clustering to assess the optimal number of clusters the cells form in each sample. Its result was two clusters, confirming the suitability of our two-cell phenotypes model. Thus, we considered a fixed number of two groups where data points were assigned according to a Gaussian Mixture Model (GMM) fitted to the data. In short, we assumed that the points were generated by two normal distributions, fitting their means and standard deviations using the maximum likelihood criterion. More specific details can be found in the documentation.

**Figure 6:**
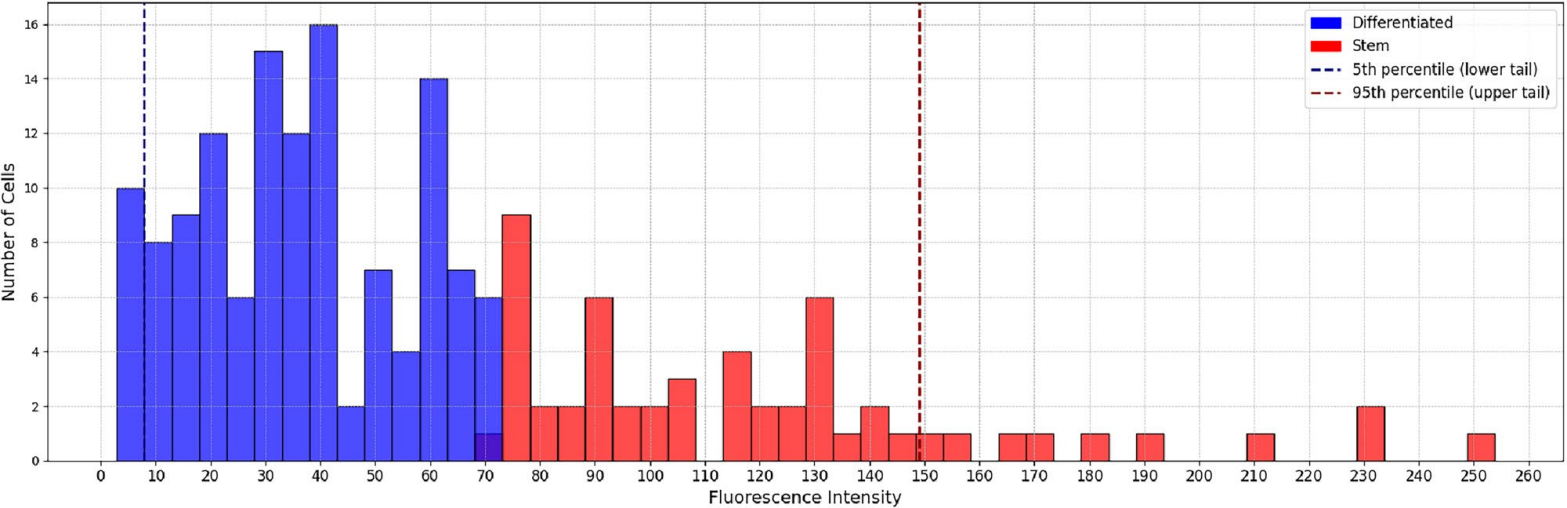
Histogram of SOX2 fluorescence intensities, colored by the GMM clustering. Intensity values were clustered into differentiated and stem cells by fitting a GMM. Regions with intensities falling in the 5th and 95th percentile were automatically considered as differentiated and stem respectively and were not taken into account when fitting the GMM.

Since the fitting of the distributions is sensitive to extreme values, the outliers, below 5th and above 95th percentiles, were directly assigned to their corresponding category, differentiated for the lowest and stem for the highest values, and not were taken into account when fitting the GMM. An example of the histogram of SOX2 fluorescence intensities for Sph1,slice3 is shown in Fig. 6. The threshold value $V=71.4$ implies that from a total of $N=183$ cells present in the spheroid, $S_V=55$ are stem cells, a fraction of $f_V\simeq 0.3$ of the total. For every case, we checked that the random seed of the clustering algorithm had not modified the threshold significantly. Usually, there are a couple of values to which the threshold converges. Checking for robustness of the clustering means that these values are close to each other, regardless of the random state used and the clustering algorithm. If these values vary a lot or are not close to each other, the clustering becomes unreliable. For the case of the example shown in Fig.6 we obtained a threshold of 71.4, for a large number of random seeds.

A more intuitive way of visualizing this result is taking Fig. 5C and coloring each region according to its clustering. This is shown in Fig. 5D where superimposed to the confocal image, the regions corresponding to stem cells are colored in red, and the regions corresponding to differentiated cells are colored in blue.

The final result of the image processing is shown in Fig. 1. There just the Voronoi regions are depicted as being colored according to their category. The image depicted in Fig. 1C corresponds to the one in Fig. 5D.

#### CSC connectedness and path forming

The location of the CSCs seems to be far from random by visual inspection of Fig. 1, since the paths that they form, break the homogeneity of their distribution. This intuition is confirmed by the graph of a *Delaunay triangulation*, the dual lattice of the Voronoi tessellation. This is nothing else than the graph given by using the centroids of the Voronoi as nodes, and adding a link between two nodes if the corresponding cells in the Voronoi diagram are neighboring cells. For instance, in Fig. 2A we depict the network corresponding to Sph1, slice3, the red and blue dots correspond to CSCs and DCCs respectively. Also we studied the subgraph with just the CSCs shown in Fig. 2B. This graph is obtained by removing all the blue nodes and all the links connected to them. We used these isolated paths, to assess if two CSCs are neighboring by chance. Particularly, we look if the nodes of the network have preferential attachment for nodes of the same type. We calculated the *homophily* of the graph, i.e. how much more likely are CSCs to be connected between them, than to DCCs. This was achieved by measuring, on the graphs, the following parameters:

- *Assortativity coefficient*: The measurement of preferential attachment that is used in network science. It is calculated as the Pearson correlation coefficient of the attribute indicating the type of cell, between pairs of linked nodes. The coefficient equals 1 for perfect assortativity (perfect preferential attachment), 0 for non-assortative networks (i.e., no preferential attachment), and – 1 for perfectly disassortative networks (perfect heterophily).
- *Homophily ratio*: Is the fraction of the edges of the graph that link nodes of the same kind. The calculation is direct, dividing the number of edges connecting cells of the same type by the total number of edges.
- *Number of stem-connected components*: At the subgraph formed by considering only the stem cells of the original graph and the links between them (stem subgraph), is the number of separated clusters. If stem cells form clusters, the number of connected components will be smaller than expected for a random distribution.
- *Degree distribution of the stem subgraph*: In the stem subgraph is the average number of stem cells that are connected to each stem cell. For instance, if the mean degree is higher than expected for randomly distributed stem cells, it means that stem cells are more connected between them than what would be expected from the random case.

A NetworkX’s^32^ implementation was used for measuring all parameters, except for the homophily ratio. For more details consult the package’s documentation).

We also look for the possibility that CSCs were placed at random positions. On the same networks obtained by Delaunay triangulation on the pictures, and leaving the connections untouched, we randomly redistributed the CSC in the graph. An example is shown in Fig. 2C where the red and blue dots are now randomly placed in other nodes, as seen when compared with panel A. In panel D we depicted the subgraph corresponding to the randomized network of panel C. Note that the number of connected components has increased from 5 in panel B to 13 for this case, a situation that occurs for most randomized networks. We performed this relocation of the whole set of CSCs 10000 times and measured the mentioned parameters joined with their corresponding standard deviations. The results comparing both, experimental and randomized networks, are reported in Table 2. We also performed statistical tests (z-test via statsmodels implementation^33^) to decide whether a given value could be obtained from the distribution of the sample, i.e. if the parameter is significantly different from the random case. In every case, we used a significance $\alpha _{t}=0.001$, but results are robust against even lower values of $\alpha _{t}$.

## Data Availability

All code and data are accessible through the GitHub repository: experimental_image_analysis.
